# The Etiology of Rachitis1Read before the Section for Clinical Medicine, Pathology, and Hygiene, of the Suffolk District Medical Society, December 9, 1885

**Published:** 1886-03

**Authors:** H. C. Haven

**Affiliations:** Boston


### SELECTIONS.

### The Etiology of Rachitis.[1]

BY H. C. HAVEN, M.D., OF BOSTON.

Although we can hardly accept Jenner's statement that rachitis is the most common, the most important, and in its effects the most fatal of the diseases affecting children, as holding true in this country, it is yet sufficiently common to make its prevention of great importance to the public health. It is, typically, a preventable disease—so far as serious results to the individual are concerned—if proper treatment is instituted early enough. It might, probably, be absolutely prevented, if we knew the ultimate causes.

[1] Read before the Section for Clinical Medicine, Pathology, and Hygiene, of the Suffolk District Medical Society, December 9, 1885.

My purpose is to briefly present the statistics I have gathered as to the effect of the commonly accepted causes of rachitis on its production in this city.

During the past three years, at the West End Dispensary for Children, there were in attendance—

| | |
|---|---|
| Total number under 7 years | 1,516. |
| " " of rachitics | 75. |
| Percentage of rachitics | 4.94. |
| Total number of colored under 7 years | 90. |
| " " " " rachitics | 38. |
| Percentage of colored rachitics | 42.79. |
| Total number of foreign ancestry under 7 years | 340. |
| " " " " " rachitics | 20. |
| Percentage of rachitics of foreign ancestry | 5.88. |
| Total number of American ancestry under 7 years | 114. |
| " " " " " rachitics | 4. |
| Percentage of rachitics of American ancestry | 3.50. |
| Number of ancestry unknown | 973. |
| " " " " rachitics | 10. |

RACE.

Classifying the rachitics as to race, we find, of the number, where it is known: Colored, thirty-eight; foreign, twenty; American, four. Of the twenty rachitics of foreign ancestry (Provinces included), there were: Portuguese, three; Italian, two; Irish, two; English, two; French, one; Nova Scotian, two; Russian, one; German one; of mixed parentage, both foreign, four; of mixed parentage, father American, two; of mixed parentage, mother American, one.

FEEDING.

| | | | | |
|---|---|---|---|---|
| Breast alone till 3 months, | 308. | 11 rachitics, or | 3.57 | per cent. |
| " " " 6 " | 295. | 7 " | 2.57 | per cent. |
| " " " 9 " | 137. | 10 " | 7.29 | per cent. |
| " " " 12 " | 200. | 7 " | 3.50 | per cent. |
| " " over 1 year | 60. | 3 " | 5.00 | per cent. |
| Breast with other food till 3 months, | 74. | 5 rachitics, or | 6.75 | per cent. |
| " " " " 6 " | 43. | 2 " | 4.65 | per cent. |
| " " " " 9 " | 83. | 3 " | 3.66 | per cent. |
| " " " " 12 " | 477. | 9 " | 1.04 | per cent. |
| " " " over 1 year, | 381. | 17 " or | 4.46 | per cent. |

| | | | |
|---|---|---|---|
| Artificial feeding only, 119. | 13 rachitics, | or 10.85 per cent. |
| Number where food not known, | | 286 |
| " " " " " | and rachitic, | 3 |

These figures need, of course, a further analysis to make them of much value, but they show, I think, very clearly, that other factors than food are, in this series of cases, the original or exciting cause of rickets.

Having noticed the frequency of rachitis among the colored in this series—and I have observed this in the attendance at other dispensaries—let us analyze the feeding of the colored children to see if, so far as they are concerned, the dictetic factor is an important one. This was as follows:

| | | | |
|---|---|---|---|
| Breast, without other food till 3 mos., | 18. | 7 rachitics, or | 30.88 per cent. |
| " " " " 6 " | 8. | 3 " | 37.50 per cent. |
| " " " " 9 " | 5. | 4 " | 80.00 per cent. |
| " " " " 12 " | 9. | 4 " | 44.44 per cent. |
| Breast with other food till 3 mos., | 7. | 1 rachitic, or | 14.28 per cent. |
| " " " " 6 " | 4. | 1 " | 25.00 per cent. |
| " " " " 9 " | 3. | 1 " | 33.33 per cent. |
| " " " " 12 " | 16. | 8 " | 50.00 per cent. |
| " " " " over 1 year, | 27. | 12 " | 44.44 per cent. |
| Artificial feeding only, | 16. | 6 rachitics, or | 37.50 per cent. |

I recognize the objection, that these statistics are too small to have great weight, but I can not but think they are sufficiently numerous to show that at least the dietetic factor is not an important one in producing rickets in colored children in Boston; the per cent. of all rachitics among all colored is only a little less than the per cent. of colored rachitics in the artificially fed from birth. And in this series of cases, moreover, many colored children are not counted as rachitic where they only showed slight bone changes. Since December 1, 1884, about the time I began to carefully note any evidence of rickets, the total number in attendance under seven years has been 495, and fifty-two colored, or 11.5 per cent.

Of the fifty-two colored, twenty-five only were non-rachitic, and twenty-seven rachitic, 51.9. This per cent. even is too small, however, I am very sure, as sometimes, in the hurry of examination, I have neglected any record as some of my charts remind me. The only question in regard to the dietetic factor yet to be spoken of, is this: Is the milk of colored mothers, otherwise healthy, as good an article of diet as the milk of the other races? I have no reason to doubt it or affirm it, except that in many of the cases there is no evident failure of nutrition on the part of the child. I hope later to present some analysis of the milk of women nursing rachitic children.

Assuming, then, the preponderance of rachitis among the colored race in Boston, and granting the effect of the diet they receive not to be a marked one, at least, as I think we are justified in doing, the question arises as to what other cause may be acting, peculiar not to any racial dyscrasia, but resulting from racial or acquired character and habits; that is, are the quarters of the city in which they live more densely crowded, are the tenements they inhabit more damp and cold or having less sunlight; are their habits of caring for their children, especially as to taking them out of doors, different from those ot the equally destitute of other nationalities? I can only give my opinion, that all these questions must be answered in the negative. I have no positive information, and hope I may obtain some from others to-night. My experience has been, that the colored are more lavish in expenditure for food and rent than other poor, taken as a class, that in equal ways their table is better equipped and the food better cooked. As for the airing of the children, I do not see how they could be taken out *less* than the children of the Irish poor, until after they can walk; after that time I have noticed no difference.

Another question must be considered. Are one or both of the parents less healthy than the average white of same social condition? If they are, this may be considersd as a causative influence, transmitted but not acting through direct heredity.

Of the 33 colored rachitics whose family history is known, both parents *said* to be healthy in 21.

Mother healthy in 4 cases; father dead in 3; I Bright's, I phthisis, I rheumatism; in one, father had cough every winter, well in summer.

Father healthy in 5; mother dead in one of phthisis; "never strong in 2;" married at 13 in I; rheumatic in I.

In 2 cases there was phthisis in father and asthma and rheumatism respectively in mother; in 1 father had phthisis and mother died in confinement.

Of 31 rachitic white, whose family history was known, parents said to be healthy in 23.

Of the 8 remaining, in 3 father healthy; mother had chorea, rheumatism, and measles at birth of child respectively.

Mother healthy in 3; father, kidney disease, arsenical poisoning, and "not strong."

Mother dead of phthisis in one, with father not strong; father rheumatic in one; mother healthy excepting catarrh which had destroyed septum, specific.

No history of syphilis in any, nor have I seen any rachitic symptoms in a number of confessedly syphilitic children.

In this comparison we see not much difference, but of course the statements of the parents are of comparatively little value; I think, undoubtedly, in this climate the average of health is less in the negro than the white native or immigrant from the north of Europe. How much lower, if any, it is than that of the immigrant from France or Italy, I have no means of forming an intelligent opinion.

In any case I do not believe there is enough difference to account for the marked preponderance of rickets on the ground of the parents' health, though it may, and undoubtedly does, exert an influence.

What, then, is the determining cause of this frequency? I see no source of any such cause but what must arise from a peculiar predisposition to this form of disease in the colored. Is this to be referred to anatomical basis, as, for instance, are the arteries of this race larger or wider? I know of no such peculiarity. And again, if the statements made to me by lay observers are true, that the disease is the less common the farther South we go, that would preclude any such hypothesis being entertained. I can find no medical testimony as to its relative frequency. It is stated, however, to be practically unknown in tropical regions. In the writings on African explorations, I have met no notice of such a disease having been noticed among the young, as it would probably have been, had it existed. Again, I have been told by lay observers that the disease is the more frequent and severe, the purer is the African blood, and I have found this to be true in my experience in this city.

The only hypothesis I can suggest to myself to account for the relative frequency of rachitics in the colored race is that as their natural habitation is much farther South than North America, the effect of the climatic conditions, which are recognized as having a causative effect in rachitics, is exaggerated; the purer the African blood the more will the effect be noticed. In other words, there is an inherited necessity for heat and sun which our climate does not satisfy, and rickets results. It is striking in this connection to notice that of the white rachitics of a foreign ancestry, the majority are born of parents coming from the south of Europe, where the disease is relatively rare, rather than of parents from England and North Germany, where the disease is rife.

I have presented these facts principally for the purpose and in the hope of eliciting criticism and information which shall enable me the better to make a more thorough study of the subject than I have had the time to do in this sketch.

